# The Impact of Microbial Composition on Postprandial Glycaemia and Lipidaemia: A Systematic Review of Current Evidence

**DOI:** 10.3390/nu13113887

**Published:** 2021-10-29

**Authors:** Megan L. Wilson, Ian G. Davies, Weronika Waraksa, Sayyed S. Khayyatzadeh, Maha Al-Asmakh, Mohsen Mazidi

**Affiliations:** 1 Research Institute of Sport and Exercise Science, Liverpool John Moores University, Liverpool L3 3AF, UK; M.L.Wilson@ljmu.ac.uk (M.L.W.); I.G.Davies@ljmu.ac.uk (I.G.D.); w.waraksa@2017.ljmu.ac.uk (W.W.); 2 Nutrition and Food Security Research Center, Shahid Sadoughi University of Medical Sciences, Yazd 8916188635, Iran; khayyatzadeh@yahoo.com; 3 Department of Nutrition, Faculty of Health, Shahid Sadoughi University of Medical Sciences, Yazd 8915173143, Iran; 4 Department of Biomedical Sciences, College of Health Sciences, QU Health, Qatar University, Doha 2713, Qatar; maha.alasmakh@qu.edu.qa; 5 Biomedical Research Center, Qatar University, Doha 2713, Qatar; 6 Medical Research Council Population Health Research Unit, University of Oxford, Oxford OX3 7LF, UK; 7 Clinical Trial Service Unit (CTSU), Nuffield Department of Population Health, University of Oxford, Oxford OX3 7LF, UK; 8 Department of Twin Research and Genetic Epidemiology, King’s College London, South Wing St Thomas’, London SE1 7EH, UK

**Keywords:** microbiota, gastrointestinal microbiome, postprandial period, dyslipidaemias, lipoproteins, lipid metabolism, lipids, glycaemic control, blood glucose, humans, animals

## Abstract

Postprandial hyperglycaemia is associated with increased risk of cardiovascular disease. Recent studies highlight the role of the gut microbiome in influencing postprandial glycaemic (PPG) and lipidaemic (PPL) responses. The authors of this review sought to address the question: “To what extent does individual gut microbiome diversity and composition contribute to PPG and PPL responses?”. CINAHL Plus, PubMed, Web of Science, and the Cochrane Central Register of Controlled Trials (CENTRAL) databases were searched from January 2010 to June 2020. Following screening, 22 studies were eligible to be included in the current review. All trials reported analysis of gut microbiome diversity and composition and PPG and/or PPL. Results were reported according to the ‘Preferred Reporting Items for Systematic Reviews and Meta-Analysis’ (PRISMA) statement. Individual microbiota structure was found to play a key role in determining postprandial metabolic responses in adults and is attributed to a complex interplay of diet, microbiota composition, and metagenomic activity, which may be predicted by metagenomic analysis. Alterations of gut microbiota, namely relative abundance of bacterial phylum Actinobacteria and Proteobacteria, along with Enterobacteriaceae, were associated with individual variation in postprandial glycaemic response in adults. The findings of the current review present new evidence to support a personalised approach to nutritional recommendations and guidance for optimal health, management, and treatment of common metabolic disorders. In conclusion, personalised nutrition approaches based on individual microbial composition may improve postprandial regulation of glucose and lipids, providing a potential strategy to ameliorate cardiometabolic health outcomes.

## 1. Introduction

Cardiovascular disease remains the leading cause of morbidity and mortality globally [1].

Characterised by hyperglycaemic spikes inducing inflammation, oxidative stress, and endothelial dysfunction, PPG is associated with the development of insulin resistance, type-2 diabetes mellitus (T2DM), and cardiovascular disease (CVD), and is considered an independent predictor of cardiovascular events [1,2]. Similarly, multiple studies have recognised PPL, which is defined as an increase in circulating plasma/serum triglyceride levels in the postprandial state, as an aetiological factor for the development of cardiovascular and chronic disease [3,4,5]. Consequently, identification of risk factors of chronic diseases is a pressing research need, with a view to improving health outcomes and alleviating the burden of disease worldwide. In previous years, traditional dietary strategies have sought to improve public health and reduce CVD prevalence globally [6]. Whilst established recommendations aim to provide guidance to much of the population, it has been argued that current guidance does not consider dietary responses on an individual level and therefore may be less effective for optimal health [7]. A growing body of evidence by recent studies suggests the role of individual characteristics in influencing metabolic responses to dietary and lifestyle factors [8,9]. A relatively new concept, personalised nutrition, refers to the tailoring of nutritional approaches to meet the needs of the individual, incorporating multiple disciplines such as genetics, epigenetics, metabolomics, and microbiology among others to determine an individual’s personal response to diet [10].

More recently, several studies have highlighted the role of metabolomics, and specifically the gut microbiome, in determining glycaemic and lipid responses, and the wider impact this may have both on health and the use of ‘omics’ technology as a viable tool to predict individual metabolic responses and personalise dietary recommendations as a result [11,12]. A complex ecosystem lives within the human gut; the microbiome is a multifunctional microbial community involved in various physiological processes including nutrient absorption, fat distribution, intestinal barrier homeostasis, and modulation of gut motility [13]. The intestinal microbiome also plays an important role in the regulation of metabolic functions, particularly maintenance of glucose and lipid homeostasis, and has been linked to various metabolic conditions including type-2 diabetes and obesity [14,15]. The extent to which the microbiota determines dietary responses is not yet fully understood; however, it is largely agreed that the gut–brain axis regulates metabolic functions through crosstalk via neural, immune, humoral, and endocrine signaling, resulting in activation of host receptors [16,17,18].

Although personalised nutrition remains a field of study in its infancy, the emergence of research within the last decade supports the concept of biological interindividual variability with studies demonstrating the variability of predictive models in determining postprandial glycaemic responses using metabolomic technology [19,20,21]. However, it is important to note that while the majority of current studies in this area analysed the effect of gut microbiota diversity on glycaemia and lipidaemia through use of animal models [22,23,24], the utilisation of human study models has increased considerably over the last decade, with studies such as Zeevi et al. (2015) [19], Berry et al. (2020) [20] and Mendes-Soares et al. (2019) [21,25] examining the extent to which individual microbiome composition affects postprandial glycaemic and lipidaemic response following consumption of standardised test meals. In addition, several studies have highlighted the role of medical intervention in modulating gut microbiota, subsequently contributing to changes in individual postprandial responses to diet (Brønden et al. (2018) [26], Mikkelsen et al. (2015) [27], and Tong et al. (2018) [28]. As such, it is of the upmost importance to provide a comprehensive overview as to the effect the microbiome has on a populational level, from both the healthy and those with pre-existing conditions.

Therefore, the aim of the current systematic review is to determine the extent to which individual gut microbiome diversity and composition contributes to inter-variability of postprandial glucose and lipid responses in human subjects based on available randomised controlled (RCTs), clinical trials, and acute experimental studies. This research also aims to elucidate the role of individual gut microbiota in predicting postprandial metabolic responses for prevention and management of glycaemia and dyslipidaemia and contribute to the discussion about whether current nutritional guidelines should be reconsidered and updated to support the current evidence. The review protocol is listed in the International Prospective Register of Systematic Reviews (PROSPERO) (CRD 42021248843).

## 2. Results

The literature search identified 22 studies (total sample size, *n* = 3851) that investigated the effect of host gut microbiota on postprandial glycaemia and/or lipidaemia either as a primary or secondary outcome. The 22 studies are summarised in Table 1.

### 2.1. Literature Search and Study Selection Process

**Figure 1:**
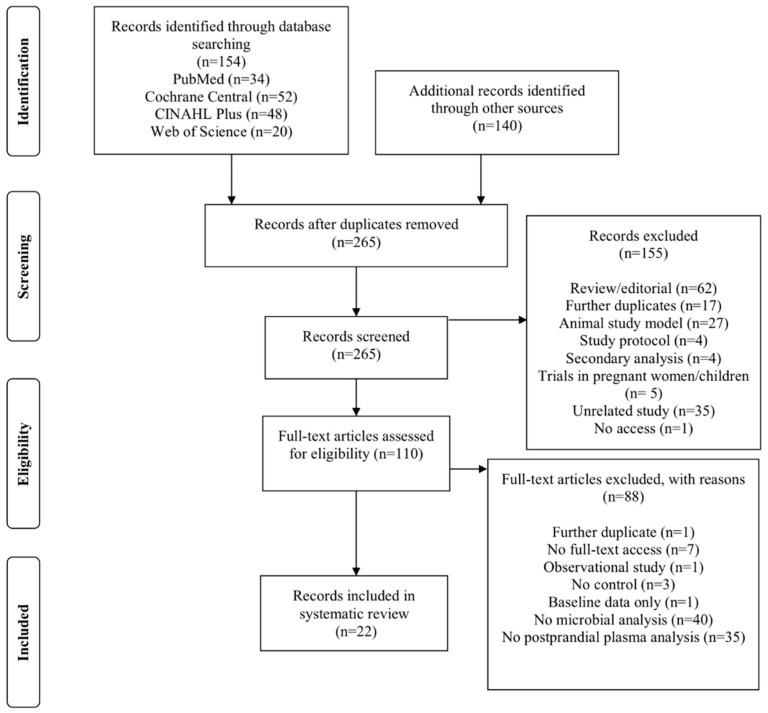
PRISMA flowchart illustrating the screening and selection process.

The study selection process that followed the literature search is summarised in Figure 1. A total of 294 citations were identified through database searches (*n* = 154) and screening of references (*n* = 140). After initial screening and removal of duplicates, 265 records remained, of which 155 were screened by abstract and excluded. Following the initial screening, 110 full-text articles were retrieved for detailed review and were assessed against the established selection criteria.

Eighty-seven full-text records did not fulfil the set inclusion criteria and after their removal, 22 studies remained eligible to be included in the current review. The reasons for exclusion of the 88 full-text articles are outlined in the methods section (Figure 1); 40 trials did not include analysis of the gut microbiome; 35 trials did not include plasma analysis in the postprandial state; 7 trials were excluded as full-text access was unavailable (abstract/protocol only); and 6 studies were not included due to lack of control, reporting on baseline results only, observational study design, and further duplication in the screening process.

### 2.2. Characteristics of the Included Studies

The characteristics of the included studies are summarised in Table 1. Published between 2010 and 2020, included studies originated from 12 countries including United States of America (five studies); China (three studies); Netherlands (three studies); Denmark (three studies); Israel (two studies); and 1 study each from United Kingdom, Spain, Sweden, Korea, Switzerland, Italy, and France. The number of participants included in the studies ranged from 5 to 1002 [21,36]. Of the 22 studies included, the current review consisted of randomised controlled trials (10), randomised trials (6) and non-randomised trials (6), of which 5 were included a series of acute experimental trials. 

With respect to the interventions that studied both diet (16 trials) and drug treatments (6 trials) to assess the extent to which the gut microbiome may be affected, studies varied from 6 days [21,25] to 17 weeks [34] in duration with participant age ranging from 18 to 80 years. Trials were conducted on both sexes, with the exception of studies by Clemente-Postigo et al. (2013) [30], Mikkelsen et al. (2015) [27], Reijnders et al. (2016) [37], and Vrieze et al. (2014) [43], which were performed only on male subjects, and Vors et al. (2020) [42], which was performed only on women. With regards to time of publication, more than 50% were published in 2018–2020. Several molecular biochemical techniques were used to assess the characterisation of the gut microbiome including DNA extraction, amplification by quantitative real-time polymerase chain reaction (PCR), Illumina [28] and whole genome 16S rRNA shotgun sequencing [25,45]. Quantification of postprandial glucose and lipid markers were assessed enzymatically or through use of a continuous glucose monitor (CGM) provided to participants for home use [46,47].

### 2.3. Modulation of the Gut Microbiome Using Dietary Intervention

Several studies highlighted the effect of dietary components on both host metabolism and gut microbial ecology [29,30,31,32,33,34,38,39,41,42]. Of the 10 trials retrieved, six studied responses to carbohydrate and protein consumption, specifically manipulation of dietary components including starch, wholegrain and gluten, as a primary or secondary outcome.

A two-arm cross-over dietary intervention by Bergeron et al. (2016) [29] compared the effects of both high and low carbohydrate diets, and high and low resistant starch (RS) intake within each arm. Separated by a 2-week washout period, the trial reported significant increases in plasma concentrations of gut microbiome-derived metabolites carnitine (*p* = 0.007), betaine (*p* = 0.008), *γ*-butyrobetaine (*p* = 0.03), and trimethylamine n-oxide (TMAO) (*p* = 0.005) after the high *v.* low-RS diet in the low-carbohydrate treatment arm in comparison to a high carbohydrate diet. In contrast, despite neither RS diet affecting fasting concentrations of insulin and plasma glucose, high-RS test meals were found to produce significantly diminished postprandial insulin and glucose responses in comparison to low-RS meals (*p* = 0.007 and *p* = 0.0001 respectively). However, no significant changes in incremental area under the curve (IAUC) were observed for PPGR following consumption of high or low-carbohydrate meals. In addition, microbial community analysis reported only correlations between relative abundance of taxa and TMAO values; therefore, it was not possible to determine the contribution of specific microbial communities impacted as a result of diet-induced changes in TMAO concentrations. However, the study noted inverse correlations between TMAO changes and two specific taxa, *Lachnospiraceae* and *Clostridiales.*

With respect to gluten intake, a cross-over trial by Hansen et al. (2018) [31] studied the effects of low vs. high gluten diets on changes in the gut microbiome in healthy individuals. The study, which consisted of two 8-week diet interventions separated by a 6-week washout period, reported alterations in relative abundance of 14 species as a result of the low-gluten diet intervention in comparison to the high-gluten diet. Specifically, both absolute and relative abundance of *Bifidobacterium ssp* were diminished during the low-gluten intervention along with several molecular pathways associated with carbohydrate metabolism: arabinose degradation, pentose phosphate pathway, phosphate acetyltransferase-acetate kinase pathway, and fructose-6-phosphate shunt. However, whilst dietary-induced modulation of the gut microbiome was observed, the study reported no significant differences in postprandial measures of glucose and lipid metabolism across interventions.

Regarding wholegrains, an earlier study by Ross et al. (2011) [38] reported no significant difference in PPL and PPG following consumption of a standardised meal despite earlier trends suggesting a decrease in fasting TC and LDL-C, but not TG, in participants consuming a wholegrain diet for 2 weeks in comparison to refined grains. In agreement, Korem et al. (2017) [32] also found no significant differences between treatments for glycaemic control andalpha or beta diversity of metagenomic species between intervention and non-intervention weeks, disproving the findings of Hansen et al. (2018) [31]. Nevertheless, Korem et al. [32] noted positive significance for relative abundance of both *Eubacterium ventriosum* species and the *Anaerostipes* genus following consumption of white bread in comparison to the sourdough intervention (*p* < 0.01). Notably, the study concluded that glycaemic response could be predicted independently using microbiome data and demonstrated highly significant interpersonal variation of glycaemic responses to both interventions (*p* < 0.05), reinforcing previous findings that metabolic responses to food are interpersonal and vary significantly between individuals [19]. In contrast, following a 4-week cross-over trial investigating the effect of consumption of whole-grain barley (WGB), brown rice (BR), and a combination of the two (WGB+BR), Martínez et al. (2013) [34] observed increased microbial diversity, Firmicutes/Bacteroidetes ratio, and faecal abundance of the genus *Blautia* following all three diet interventions. Regarding glucose and lipid metabolism, the study reported improvements in both fasting and postprandial peak glucose levels, the latter of which reached significance in overweight subjects during the combined WGB and BR period (*p* < 0.05); a significant reduction in both TC and LDL-C concentrations was also observed (*p* < 0.05 for both).

Upon further analysis of sequence data, Martínez et al. (2013) [34] found that consumption of WGB enriched the genera *Roseburia*, *Bifidobacterium,* and *Dialister* along with species *Eubacterium rectale* (*E. rectale*), *Roseburia faecis*, and *Roseburia intestinalis*. In addition, following treatment with WGB and BR, both peak postprandial glucose plasma and interleukin-6 (IL-6) decreased; conversely, *Coriobacteriaceae* abundance significantly decreased and proportions of *Dialister* increased in subjects with the highest improvement in IL-6. Moreover, proportions of Bacteroidetes, *Bacteroidaceae,* and *Bacteroides* were positively correlated to plasma HDL-C values (*R* = 0.54–0.56, all *p* < 0.05). The study concluded that diet-induced increases in the abundance of *E. rectale* was associated with improvements in postprandial glucose and insulin response in normal and overweight subjects. In contrast, Schutte et al. (2018) [39] investigated the benefits of whole-grain wheat (WGW) in comparison to refined wheat (RW) consumption in overweight subjects. Discordant with the earlier findings of Ross et al. (2011) [38], the trial reported significant differences in PPL concentration between intervention groups (*p* = 0.020), with postprandial TG levels peaking significantly at the 4 h timepoint in the WGW group (*p* = 0.004). Moreover, significant alterations in intestinal microbial diversity were noted between the WGW and RW group (*p* = 0.035); however, specific changes in species abundance were not observed between groups. Similarly, Kovatcheva-Datchary et al. (2015) [33] examined the effect of barley kernel-based bread (BKB) and white wheat flour bread (WWB) on gut microbiota and glucose metabolism in healthy participants. Following administration of a standardised test meal, mean PPG and serum insulin responses improved following 4-day consumption of the BKB in comparison to WWB (iAUC 0–150 min). On a microbial level, Prevotella/Bacteroides ratio increased after BKB consumption in responding individuals with further analysis indicating enriched abundance of *Prevotella copri*, though this change was only noted in individuals considered more responsive to the intervention.

Considered a modulator of the gastric microenvironment, alcohol use is associated with the breakdown of the gastric barrier, impaired insulin secretion, and increased circulating plasma triglycerides [48,49]. A study by Clemente-Postigo et al. [30] analysed the effect of chronic and acute alcohol consumption and high fat intake on plasma lipopolysaccharides (LPS) concentrations in middle-aged men. Following the consumption of red wine, dealcoholized wine, or gin, combined with a dietary fat overload, the study found no significant changes in postprandial TG or postprandial chylomicron LPS concentrations between treatments, which are calculated as the difference between postprandial and baseline values. In contrast, a positive correlation was identified for postprandial chylomicron LPS concentrations and changes in TG (*R* = 0.517, *p* = 0.028). Conversely, increased abundance of Firmicutes, Bacteroidetes, and Proteobacteria phyla were also observed following red wine consumption. However, it is important to note that small sample size (*n* = 10) was identified as a limitation to the postprandial study and may have limited the significance of findings.

Finally, two studies investigated the impact of dietary fat consumption on postprandial metabolic response and intestinal microbial diversity. Vetrani et al. (2020) [41] evaluated the effects of diets rich in polyphenols (PP) and/or long-chain n-3 polyunsaturated fatty acids (LCn3) on intestinal microbiota composition in subjects with high cardiovascular risk. The study found that diets rich in PP increased bacterial diversity, whilst diversity diminished following consumption of both diets low in LCn3 and PP, and diets high in LCn3 respectively. In addition, a significant correlation was observed between changes in the *Atopobium* cluster and large VLDL triglycerides (*R* = 0.313, *p* = 0.009), cholesterol in large VLDL (*R* = 0.319, *p* = 0.008), and postprandial triglycerides in plasma (*R* = 0.266, *p* = 0.026). In comparison, a similar study by Vors et al. (2020) [42] examined the effect of milk polar lipid (PL) consumption on lipid metabolism, absorption, microbiota, and known markers of cardiometabolic health in a population of postmenopausal women. Following 4-week consumption of 0, 3, or 5 g PL/day, the study reported significant reductions in both fasting and postprandial concentrations of serum cholesterol with analysis showing significant alterations between groups for measures of TC, TG, apolipoprotein B (ApoB), and apolipoprotein A1 (ApoA1) (*p* _posthoc_ < 0.05 vs. control). Conversely, it is important to note that a non-significant decrease in postprandial TG was only observed in the group consuming 5 g-PL/day. Subsequently, Vors et al. [42] concluded that daily consumption of milk polar lipids did not result in significant modulation of either phylogenetic groups or bacterial species of gut microbiota.

### 2.4. Individual Intervariability of Metabolic Responses and Microbial Diversity Using a Standardised Approach

Five studies investigated the extent to which individual gut microbiome composition contributes to metabolic responses through analysis of a series of acute experimental trials, of which all noted high interpersonal variability in postprandial response following consumption of standardised test meals (Table 1) [19,20,21,25,40]. Adopting a similar methodological framework to that of Zeevi et al. (2015) [19], a number of trials concluded substantial variations in glucose responses following consumption of identical meals, with one study reporting glycaemic excursions ranged from 6–94 mg/dL (0.3–5.2 mmol/L) above baseline following consumption of a standardised meal (0–150 min) [21]. Two studies by Mendez-Soares et al. (2019) [21,25] analysed the extent to which individual postprandial responses could be reproduced in a Midwestern USA cohort, building on the previous model created by Zeevi et al. (2015) [19]. Utilising stochastic gradient boosting regression to predict PPGRs, both studies found that, despite differences in population characteristics, the framework model of Zeevi et al. [19] accurately predicts PPGRs when tested on a Midwestern cohort consuming a Western diet, outperforming traditional methods used to inform dietary interventions to better regulate glycaemic control in a cohort of 327 and 297 healthy, non-diabetic individuals, respectively.

Most notably, both studies observed significant variability and interindividual reproducibility of individual glycaemic responses to dietary carbohydrate and related sensitivity. As predicted, carbohydrate amount was positively associated with PPGRs with the subsequent study reporting 93.5% of participants in the cohort exhibited a positive correlation between the amount of carbohydrates consumed in a single meal and PPGR in comparison to 98% of participants in the Israeli cohort observed by Zeevi et al. (2015) [19,25]. In addition, the second study noted significant variability in the above correlation with participants showing both high and low carbohydrate sensitivity in response to identical meals, supporting the previous findings of the earlier study by Zeevi et al. (2015) [19], highlighting the extent to which inter-individual characteristics contribute to variability across individuals. The latter study also compared species richness and diversity between cohorts, noting a significantly lower microbiome diversity in the Midwestern cohort than the Israeli cohort, which is attributable to population characteristics between cohorts such as fibre intake. Additional correlations were identified including lower microbial diversity in overweight and obese subjects (*p* < 0.05) in comparison to individuals of a normal weight. In relation to microbiome-related outcomes, Mendez-Soares et al. (2019) [21] reported an inverse relationship between *Eubacterium rectale* and PPGR values, and a significant decreased abundance of *Prevotella/Bacteroides* ratio was also observed in the Midwestern USA cohort (*p* < 0.05), consistent with reduced fibre consumption associated with a standard Midwestern diet.

The previous findings are in agreement with a 2020 validated cohort study by Berry et al. [20], which reported considerable heterogeneity across all postprandial time points for glucose, insulin, and triglyceride concentrations, following a standardised meal test. The findings of which thereby strengthen the earlier conclusions of Tily et al. (2019) [40], reiterating the widely accepted hypothesis that glycaemic response is driven by both individual characteristics and the macronutrient content of foods when measured using multiple diet types and populations.

### 2.5. Interaction between Drugs and the Gut Microbiome

We found seven trials in the past decade (Table 1) that investigated the effect of drug administration, medicinal or probiotic supplementation on postprandial responses and microbiota profile [26,27,28,36,37,43,44]. All seven studies compared results with baseline measurements prior to undertaking the intervention. Three trials studied the effects of antibiotic use [27,37,43], another one the use of sevelamer [26], one with the use of probiotics [36], and two measured the effects of supplementation with traditional Chinese herbal formulas, one of which also included analysis of an antidiabetic medication [28,44].

For studies assessing the effects of gut microbiota manipulation by antibiotics on host metabolism, two trials compared treatment of amoxicillin, vancomycin, and placebo for a duration of 7 days in overweight or obese male subjects with metabolic syndrome (MetS) or impaired glucose tolerance (IGT) (Table 1) [37,43]. Reijnders et al. (2016) [37] reported significantly decreased relative abundance of bacteria primarily of the Firmicutes phylum (*p* = < 0.001) and increases in gram-negative Proteobacteria, certain species of *Clostridium* cluster IX, and Bacilli including *Lactobacillus plantarum* and *Enterococcus* following treatment of 500 mg vancomycin three times daily in comparison to groups treated with amoxicillin or placebo at the same dose. Similarly, Vrieze et al. (2014) [43] concluded treatment with vancomycin significantly decreased gut microbial diversity in comparison to amoxicillin (*p* < 0.01), citing Firmicutes phylum, specifically *Clostridium* cluster IV and XIVa, *Lactobacillus plantarum*, *Faecalibacterium prausnitzii,* and *Eubacterium hallii* as species affected by vancomycin, although a non-statistically significant trend indicative of increased microbial diversity was identified in the amoxicillin group. Comparably, both studies reported non-significant results for postprandial plasma glucose, TG, or FFA concentrations in either treatment group despite aberrant vancomycin-induced changes in gut microbiota.

Moreover, a 2015 study by Mikkelsen et al. [27] reported a decrease in total anaerobic bacterial count, markedly species of *Enterococci*, *coliforms,* and *Bifidobacterium*, after 4 days of treatment of an oral antibiotic cocktail comprised of 500 mg vancomycin, 40 mg gentamycin, and 500 mg of meropenem once daily in lean, glucose-tolerant males. However, despite substantial growth and colonisation of both aerobic and facultative anaerobic bacteria, no change in glucose tolerance, measured by the total area under the curve (tAUC_glucose_), was observed between baseline, immediately post-intervention, or during follow-up testing (42 days). In contrast, Brønden et al. (2018) [26] found that the use of sevelamer, a phosphate-binding drug typically used to treat hyperphosphatemia in patients with chronic kidney disease, significantly improved postprandial glucose excursions and increased β-cell function (HOMA-β) (*p* = 0.01 and *p* = 0.03 respectively). Interestingly, the study reported no significant changes in gut microbiota species richness following treatment of the drug when tested in subjects with T2DM.

An alternative approach using traditional Chinese medicine (TCM) to modulate gut microbiota for the alleviation of T2DM has been investigated by two studies [28,44]. A positive-control clinical trial by Tong et al. (2018) [28] compared the effects of AMC, a traditional Chinese herbal formula, and metformin, an anti-hyperglycaemic drug used in the treatment or prevention of diabetes and hyperlipidaemia. After 12 weeks of treatment, both metformin and AMC significantly improved 2-h PPG (*p* < 0.0001) and altered the gut microbiota structure of T2DM patients when analysed by principal-component analysis (PCA) and principal-coordinate analysis (PCoA) based on Bray Curtis dissimilarity [35]. In addition, treatment with AMC significantly improved TG levels but decreased microbiome diversity as indicated by a significant reduction of Chao1 richness (*p* < 0.01 and *p* < 0.001 respectively); however, the status during which plasma lipid samples were collected is unclear from the methods provided. Similarly, Xu et al. (2015) [44] examined the effects of Chinese herbal formula GQD on short-term manipulation of gut microbial dysbiosis in subjects with T2DM. The study, a 12-week randomised controlled trial, reported significant improvements in glycaemic control and 2-h PPBG (*p* < 0.001), along with alterations of gut microbiota structure after 4 weeks of treatment in comparison to baseline and placebo.

In contrast, a recent study by Park et al. (2020) [36] highlighted the effect of probiotic supplementation in ameliorating LDL-C and postprandial lipid biomarkers following 12 weeks of probiotic treatment with *Lactobacillus plantarum* Q180. The area under the curve (AUC) and maximum concentration (C_max_) of postprandial TG significantly improved in those treated with LPQ180 in comparison to placebo (*p* = 0.049 at 6 h); maximum concentration of chylomicron (CM) TG also decreased significantly (*p* = 0.020). Conversely, Park et al. (2020) [36] reported no significant changes in intestinal flora diversity between placebo and LPQ180 groups over the 12-week period. However, non-significant changes in TC, TG, and phenol levels in the LPQ180 group were negatively correlated with baseline abundance of *Ruminococcus bromii*. Likewise, positive changes in TC and LDL-C were associated with increased baseline levels of bacterium *Kineothrix alysoides* in individuals in the LPQ180 group.

## 3. Discussion

To our knowledge, this is the first systematic review that compiles and provides effects and associations of gut microbiota composition and postprandial glycaemia and lipidaemia in human subjects. Despite heterogeneity across trials, the findings of the 22 included studies, summarised in Table 1, indicate an association between gut microbial composition and individual postprandial glycaemic and lipidaemic responses in both healthy adults and individuals with pre-existing conditions including MetS, T2DM, and obesity, which are key risk factors of cardiovascular disease [50].

The gut microbiota has been shown to play a critical role in human health, particularly the development of metabolic diseases [51], and is considered a co-determinant of postprandial plasma glucose response [52]. Given that humans spend a significant proportion of time in a postprandial state, it is of the upmost importance that underlying mechanisms influencing metabolic responses post-consumption are studied in greater detail [53]. The relationship between the gut microbiota and metabolism has gained prominence in the last two decades with methods for analysis of microbial composition advancing rapidly within the last 15 years [54,55]. However, the extent to which individual microbiota structure plays a role in determining glycaemic and lipidaemic responses to dietary intake is lesser known, with machine-learning models for predicting metabolic responses remaining an emerging area of study [20]. Largely modulated by diet in humans, the genetic composition and metabolic activity of intestinal microbiota has been shown to respond rapidly to changes in dietary intake, with inter-individual variations in microbial gene expression and the diversity of human diet and lifestyle attributable to dietary factors [56].

Based on 17 included studies, our findings of interventional trials yielded discordant results, largely due to methodological differences and contrasting clinical outcomes. Findings from dietary interventions suggest the consumption of high resistant starch (RS) and carbohydrate (CHO) diets upregulate circulating levels of trimethylamine n-oxide (TMAO), a gut-derived metabolite associated with multiple inflammatory diseases including atherosclerosis [25,57]. However, as expected, meals high in RS and wholegrains improved PPGR in comparison to refined counterparts, thereby improving CVD risk through improved carbohydrate metabolism. Notably, a 2016 study by Bergeron et al. [29] found that consumption of meals providing 16–22 g of RS significantly attenuated both postprandial insulin and PPGR in individuals. This is consistent with the findings of a 2017 systematic review and meta-analysis by Marventano et al. (2017) [58], which attributed improved postprandial glucose excursions with the fermentation of indigestible polysaccharides by the microbiota, resulting in the production of short-chain fatty acids (SCFA), improved glucose oxidation, and increased insulin sensitivity. In addition, dietary consumption of whole grains is associated with compositional alterations of the gut microbiota, namely diet-induced increases of butyrate-producing *Eubacterium rectale* and the genus *Prevotella*, a leading source of inter-individual microbiota variation [59], which has been reported to improve glucose tolerance and influence glucose homeostasis via intestinal gluconeogenesis [33,60,61]. However, several studies failed to report the significance of either diet-induced modulation of metabolic responses, microbiome composition, or metagenomic activity [26,36,42], which is likely attributable to limitations such as small sample size and/or short study duration.

Conversely, our results of drug intervention trials suggest strong effects of antibiotic, hyperphosphatemia, and hyperglycaemia medication on modulation of the gut microbiota, consistent with existing literature in this field [62,63,64]. Nevertheless, positive alterations of postprandial glucose or lipid metabolism were not observed following treatment of antibiotics, despite significant changes in microbiota structure. Lack of clinically relevant associations between significant changes in gut microbiota and metabolic parameters is in contrast to the current dogma, which emphasises the role of gut microbiota as a key component involved in metabolism regulation [27,65]. It is important to note that almost half of the drug intervention studies had a relatively short duration of 7 days, which may be one reason why they showed no significant effect on metabolic parameters [27,35,44].

In contrast, whilst the majority of studies reviewed sought to establish a cause-and-effect relationship between alterations in gut microbiota and improved postprandial glucose and lipid responses, population-based cohort studies indicate associations with gut microbiota, and metabolic responses are largely specific to the individual [19,20,21,25,40]. Substantial intra-individual variability was observed across all cohort studies and has been attributed to multiple features of the microbiome. Through analysis of microbiome profiling and utilisation of predictive models, several associations were reported between functional properties of microbiota and variability in postprandial glucose responses in humans. For instance, in one of the earlier studies to identify major factors predictive of PPGRs, Zeevi et al. (2015) [19] observed associations between taxa such as Actinobacteria, Proteobacteria, and Enterobacteriaceae with dietary habits and various phenotypes related to obesity and glycaemic control, along with identification of lesser-known functional pathways, believed to be a result of variation of bacterial taxa amongst individuals [19,66]. Moreover, building on the previous findings of Zeevi et al. (2015) [19], Berry et al. (2020) [20] further quantified the extent to which the gut microbiome contributes to metabolic responses, elucidating that microbiome composition accounted for 7.5% of postprandial triglyceride (6 h) and 6.4% of postprandial glucose (IAUC, 0–2 h) independent of individual characteristics. Additional meal-related factors including macronutrient composition and individual meal-specific responses were also found to be more predictive of PPGR than previously thought with genetic parameters found to be less influential in comparison to meal timing in determining lipidaemic response. In contrast to existing literature, the principal findings of Berry are in disagreement with previous studies [40,67,68], which largely attributes metatranscriptomic activity of the gut microbiome as the key determinant of individual postprandial glycaemic response.

Upon analysis, further mechanisms associated with the gut microbiome, host metabolism, and the ability to predict PPGRs include fucose metabolism, indoleacetate and glutamine production pathways, and fructose and tyrosine metabolisers [40]. However, Tily et al. (2019) [40] noted that more research is needed to establish the exact mechanisms connecting the metabolism of gut sugars and increased PPGRs in individuals due to the complexity of the mechanisms involved in microbial metabolic regulation.

Despite high levels of intra-individual variability in PPGR and PPL responses, which are attributed to a complex interplay of diet, microbiota composition, and metagenomic activity, the findings of the current review reinforce the hypothesis that once postprandial responses to specific foods are established, metabolic response to other dietary components may be accurately predicted. However, it is important to acknowledge that further research is needed to better understand the role of individual microbiota and improve homogeneity across studies, allowing for more in-depth analysis of data such as baseline and endpoint PPGR and PPL response. Further analysis of the specific nutritional breakdown included in standardised test meals would also be beneficial in order to accurately develop tailored nutritional approaches, paving the way for precise personalisation of diet recommendations [23].

## 4. Materials and Methods

### 4.1. Literature Search Strategy

The current study sought to conduct a systematic review on the contribution of the individual microbiome composition on postprandial glycaemia and dyslipidaemia in humans, and the extent to which interpersonal variability of the microbiome contributes to the predictability of glycaemic and lipidaemic control. We also gave attention to the effectiveness of analytical models in predicting postprandial metabolic responses in human subjects. This systematic review was performed and reported according to the Preferred Reporting Items for Systematic Reviews and Meta-Analysis (PRISMA) statement [69] and PICOS (Population, Intervention, Comparison, and Outcomes) [70] criteria used to define the following research question: To what extent does the individual gut microbiome diversity and composition contribute to the variability of postprandial glycaemia and dyslipidaemia in human subjects? (Table 2).

### 4.2. Search Methods

CINAHL Plus, PubMed, Cochrane Central, and Web of Science were searched for relevant randomised trials published between January 2010 and June 2020 with no restriction on language. Databases were searched individually with advanced search strategies utilising various combinations of controlled phrases as either keywords or MeSH terms. To maximise search sensitivity, we combined multiple terms relating to both the gut microbiome and postprandial state in addition to glycaemia and lipidaemia to enhance precision (Supplementary Material). The wild-card term “*” was included to increase sensitivity of the search strategy.

Following the initial database search and screening, full-text articles were independently reviewed by two of the authors to ensure all studies included were relevant and met the inclusion criteria for the current study (W.W. and M.W.). Furthermore, to minimise the effect of publication bias, a snowball method, characterised by manual checking of references from retrieved articles, was applied to relevant studies that met the selection criteria outlined below.

### 4.3. Selection Criteria

The current review included all clinical trials and non-randomised acute experimental studies that evaluated the effect of the individual gut microbiome diversity on postprandial glycaemia and lipidaemia. Eligible studies were screened to meet the following criteria: (1) adult study population (aged 18+); (2) randomised controlled or clinical trials with either parallel, crossover, or acute experimental studies (a series of non-controlled acute studies that tested microbiome, glycaemic, and lipidaemic responses to various test meals on different days in large cohort populations); (3) use of dietary or pharmacological intervention; (4) presence of both metagenomic and postprandial plasma analysis; and (5) presentation of sufficient information on primary objective/outcome at baseline and endpoint or provision of net change values. Exclusion criteria was as follows: (1) non-clinical studies; (2) animal and/or in vitro study models; (3) pregnancy/child studies; (4) studies that did not provide baseline or endpoint values for outcomes of interest; and (5) narrative reviews, opinion pieces, editorials, protocols, and/or studies that did not include primary data were also excluded from the review.

### 4.4. Data Extraction and Critical Appraisal

Duplicate studies were removed, and remaining studies screened by title and abstract initially before full-text articles were reviewed by two researchers independently (M.W., W.W.) to minimise risk of bias. Following the initial screening, studies were listed as either included, excluded, or pending if the eligibility of the study to be included in the review was unclear. Pending studies were temporarily included in the next stage of screening. Once retrieved, full-text articles were independently reviewed, and inclusion/exclusion criteria applied. Discrepancies in the selection process were resolved at a meeting between reviewers prior to the retrieval of the final selection of papers. Based on the PRISMA guidance, a flowchart was produced to enable transparency of the screening process (Figure 1).

### 4.5. Quality Assessment

A systematic assessment of bias in the included studies was performed in accordance with the criteria established by Cochrane [71]. Studies were assessed on the following: adequacy of random sequence generation, blinding of participants, personnel and outcomes assessment, selective outcome reporting, allocation concealment, and handling of dropouts (incomplete outcome data). Discrepancies regarding the presence of bias were resolved by discussion with a second and third author (M.M and I.G.D) to resolve inconsistencies and reach a consensus.

### 4.6. Data Extraction and Management

Full-text studies that met the inclusion criteria were retrieved and screened to assess eligibility by two reviewers (M.W., W.W.). Once methodological quality was determined, the reviewer (M.W) extracted and transferred to a Microsoft Excel spreadsheet and briefly summarised key concepts, findings, and results from each study. Summaries for each study were discussed with a third reviewer (M.M.) and any inconsistencies resolved. Data was organised by first author, year of publication, country of origin, age range and gender of participants, study design and duration, intervention type, presence of background disease/conditions, and summary of key findings (Table 1).

### 4.7. Assessment of Risk of Bias

**Figure 2:**
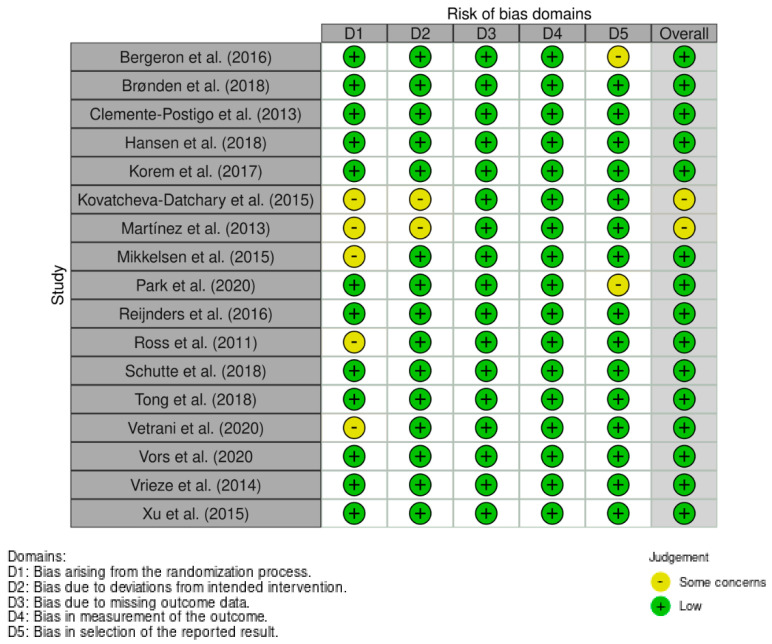
Quality assessment of included randomised trials (*n* = 17) using the Cochrane Risk of Bias Tool.

The quality and the risk of bias across all included studies were assessed using the Cochrane Risk of Bias Tool [71] (Figure 2). Alternatively, studies containing a series of acute experimental trials were assessed using the (ROBINS-I) tool [72]. Due to the nature of several studies, randomisation and blinding of participants and/or outcome assessors was not possible; however, as all outcome measures were objective, it was determined that it was unlikely that this influenced the results of the studies. For studies that utilised a series of acute experimental trials [21,28,41,46,51], risk of bias was assessed separately in accordance with the recommended guidance provided by Cochrane regarding assessment of bias risk in cohort studies [71]. Overall, no study included in the review received a “high” risk of bias result in any assessed category.

## 5. Strengths and Limitations of the Current Review

To our current knowledge, this is the first systematic review to comprehensively address the impact of individual gut microbiota in influencing metabolic parameters attributed to postprandial hyperglycaemia and lipidaemia in human subjects. The use of multiple databases and manual searching of reference lists associated with key studies was beneficial in maximising access to trials that encompassed a broad spectrum of population and methodological considerations whilst meeting strict inclusion and exclusion criteria, allowing for effective identification of comparisons and contrasting aspects between studies. In addition, whilst the study allowed for a wide range of participants including both healthy individuals and those with chronic disease, we acknowledge that different baseline characteristics can influence microbiome diversity, and therefore cannot be considered comparable. Given the limited number of papers on individual postprandial glycaemic/lipidaemic response and gut microbiome diversity, the inclusion criteria was designed to include all available studies to provide a broad comprehensive review. The presented study had several limitations; it is important to note that due to the presence of considerable clinical, methodological, and statistical heterogeneity across studies, a statistical meta-analysis was not performed. This was determined as substantial methodological heterogeneity; contrasting outcome measures and metrics were inconsistent throughout the final included studies. In addition, sample size and study duration varied significantly across studies, ranging from 5 to 1002 participants and 7 to 180 days respectively, a factor which may have limited the statistical power of the included studies and potentially compromised research outcomes. Moreover, whilst all studies included postprandial plasma and microbiome analysis, interventions varied across trials and included both dietary and pharmacological interventions, which is important to note due to the complexity of diet/drug interactions, how such interactions influence the microbiome, and consequent contribution to alterations in PPGR and PLL responses.

## 6. Conclusions

The findings of the current review present new evidence to support a personalised approach to nutritional recommendations and guidance for optimal health, management, and treatment of common metabolic disorders. However, the mechanisms of microbial metabolic regulation remain unclear due to the complexity of the microbiota. These findings provide greater insight for future investigation of the complex interplay between diet, gut microbiota, and postprandial metabolic responses that may support the development of tailored nutritional approaches for preventing cardiometabolic disease in humans. In conclusion, a personalised approach to nutrition based on individual microbial diversity may improve postprandial regulation of plasma glucose and lipids. However, further research is warranted to objectively measure the extent to which individual gut microbiome diversity and composition contribute to the variability of postprandial glycaemia and dyslipidaemia in humans, and further support the concept of personalised nutrition as an effective alternative to traditional strategies for management and treatment of metabolic disorders in individuals. 

## Figures and Tables

**Table 1:** Characteristics of included studies.

First Author, Year, Country	Study Design	Age Range (year)	Male (%)	Background Disease	Sample Size (*n*)	Study Duration	Intervention Type	Summary of Key Findings
Bergeron et al. (2016), USA [29]	RCT	≥20 y	38.5	Healthy men and post-menopausal women with no history of CVD or other chronic diseases.	52	8 weeks	Diet: High and low total CHO intake compared to high vs. low resistant starch intake.	High RS intake significantly increased circulating levels of trimethylamine n-oxide (TMAO) (*p* < 0.0001). Higher-CHO diets increased plasma TG and large VLDL-C particle concentrations and promoted a shift in LDL-C particle distribution towards increased medium and small LDL-C independent of starch digestibility. High RS test meals significantly lowered PPGR and insulin responses in comparison to low RS meals (*p* < 0.0001 and *p* = 0.007 respectively). *Lachnospiraceae* and *Clostridiales* inversely correlated with TMAO change. Conclusion: High RS meals, not diets, produced significantly lower postprandial insulin and glucose responses.
Berry et al. (2020), UK [20]	Series of acute experimental studies	18–65 y	27.8	Healthy subjects with no history of chronic diseases.	1002	2 weeks	Diet: Standardised meal testing to measure and predict individual metabolic responses.	Heterogeneity across all postprandial time points (during fasting for 6 h) varied greatly for triglyceride (*p* = 3.931 × 10^–11^), glucose (*p* = 2.91 × 10^–194^), and insulin (*p* = 2.45 × 10^–17^) concentrations. High inter-individual variability was observed in PPL, PPG, and insulin following consumption of standardised meals. Gut microbiome composition (independent contribution) explained 7.5% of PPL (6 h) and 6.4% of PPGR (IAUC 0–2 h). Conclusion: Gut microbiome composition had a greater influence than macronutrient content of meals for PPL but not PPG.
Brønden et al. (2018), Denmark [26]	RCT	35–80 y	70	Healthy subjects and patients with T2D for at least > 3 months.	30	7 days	Drug: Treatment of 1600 mg of sevelamer or placebo.	PPG excursions significantly reduced in sevelamer treatment group. Trend for increased basal and postprandial plasma cholecystokinin following treatment of sevelamer in comparison to placebo. No significant changes in gut microbiota species richness or overall composition in comparison to placebo. Conclusion: Treatment with sevelamer reduces PPG concentrations in individuals with T2D.
Clemente-Postigo et al. (2013), Spain [30]	RCT	45–50 y	100	Healthy subjects with no history of chronic diseases.	5	12 days	Diet: 4-arm crossover intervention: 50 g fat overload, red wine/fat overload, dealcoholised red wine/fat overload, and 100 mL gin/fat overload.	Changes in TG concentrations (calculated as the difference between postprandial and baseline values) were not significantly different between treatments. Concentrations of postprandial LPS did not differ between treatment groups. Consumption of a high-fat meal resulted in higher postprandial LPS concentrations. Conclusion: chronic red wine consumption modifies gut microbiota by increasing different bacterial phyla such as *Firmicutes*, *Bacteroidetes*, *Proteobacteria*, and *Actinobacteria*, resulting in lower LPS concentrations in comparison to acute intake of red wine only.
Hansen et al. (2018), Denmark [31]	RCT	18–65 y	42.6	Obesity/Risk of MetS.	60	16 weeks	Diet: Low or high-gluten diet in comparison to habitual intake.	An 8-week low-gluten diet intervention induced changes in the gut microbiome and fermentation of complex CHO in healthy adults. No effects on glucose or lipid metabolism were noted for either intervention groups. Conclusion: Consumption of a low-gluten diet induced microbial changes in the gut but did not significantly improve glucose or lipid metabolism in obese individuals at risk of MetS.
Korem et al. (2017), Israel [32]	Randomised crossover trial	18–70 y	55	Healthy subjects with no history of chronic diseases.	20	2 weeks	Diet: Consumption of sourdough bread compared to industrially made white bread.	No significant difference in glycaemic control between treatment groups. Significant increase in relative abundances of *Eubacterium ventriosum* species and *Anaerostipes* genus following white bread consumption compared to sourdough. No significant differences reported for α-diversity or functional properties of gut microbiome. Bread consumption regardless of type significantly decreased total cholesterol and LDL-C but not HDL-C levels. Significantly higher variance in PPGR noted both for individuals on standardised meals and combined diets. Conclusion: individuals exhibit significant alterations in gut microbiota and personalized PPGRs to bread regardless of type.
Kovatcheva-Datchary et al. (2015), Sweden [33]	Randomised crossover trial	50–70 y	15.4	Healthy subjects with no history of chronic diseases.	39	3 weeks	Diet: Consumption of barley kernel-based bread (BKB) or white wheat flour bread (WWB).	Mean PPG and insulin responses after a standardised breakfast were improved following BKB compared with WWB in the total group (*p* < 0.01 at 30- and 45-min timepoints). *Bacteroidetes* abundance increased following BKB consumption in the ‘responder’ group but not the non-responders. Repetition of the study 12 months later observed improved PPGR after BKB intervention in ‘responding’ subjects, indicating stability of the microbiota over time. Conclusion: BKB-induced increases of *Prevotella* are associated with improvements in glucose tolerance in responsive subjects.
Martínez et al. (2013), USA [34]	Randomised crossover trial	18–65 y	39.3	Healthy subjects with no history of chronic diseases.	28	17 weeks	Diet: Consumption of 60 g of whole-grain barley, brown rice, or an equal mixture of the two.	Microbial diversity increased across all treatments. Consumption of 60 g whole-grain barley enriched the genera *Roseburia*, *Bifidobacterium,* and *Dialister*, and the species *Eubacterium rectale*, *Roseburia faecis,* and *Roseburia intestinalis*. Whole-grain barley and combination treatment reduced plasma IL-6 and peak PPGR. Changes in abundance of *Eubacterium rectale* were associated with changes in the glucose and insulin postprandial response. Conclusion: short term intake of whole grains induced alterations of the gut microbiota that coincide with improvements in measures related to metabolic dysfunctions in humans.
Mendes-Soares et al. (2019), USA [25]	Series of acute experimental studies	≥18 y	23	Healthy subjects with no history of chronic diseases.	297	6 days	Diet: Standardised meal testing to measure and predict individual metabolic responses.	Significant variation in dietary CHO intake was indicative of the complexity of predicting glycaemic responses due to individual variability within the sample. Relative abundances of Actinobacteria, *Eubacterium eligens*, *Alistipes putredinis*, and *Subdoligranulum* showed mostly a direct relation to PPGR, agreeing with the findings of Zeevi et al. (2015)^52^. Replicating the methodology of Zeevi et al. (2015)^52^, the study found that the model used to predict individual PPGR could be modified for use in other populations (Midwestern US cohort). Conclusion: A predictive model to anticipate personalised metabolic responses to dietary intake outperforms previous common approaches used to inform dietary interventions to regulate glycaemic control.
Mendes-Soares et al. (2019), USA [21]	Series of acute experimental studies	≥18 y	22	Healthy subjects with no history of chronic diseases.	327	6 days	Diet: Consumption of two different standardised test meals (plain bagel with cream cheese and cereal with or without milk) to measure and predict individual metabolic responses.	Utilised the modelling framework used by Zeevi et al. (2015) [35]. Postprandial glucose response varied substantially across participants with glycaemic excursions ranging from 6–94 mg/dL or (0.3–5.2 mmol/L) above baseline following consumption (0–150 min). The study noted significant intraindividual reproducibility of glycaemic responses to the standardised meal. In comparison to a subset of non-diabetic participants from the Israeli cohort (Zeevi et al. 2015) [35], α-diversity was significantly lower in the Midwest cohort. Microbiome analysis observed decreased abundance of *Actinobacteria* and *Prevotella*/*Bacteroides* ratio, and increased *Firmicutes/Bacteroidetes* ratio observed in the Midwestern cohort in comparison to the Israeli subset. Conclusion: A model for predicting personalised responses to dietary intake can predict individual glycaemic responses to a range of diverse foods.
Mikkelsen et al. (2015), Denmark [27]	Clinical trial	18–40 y	100	Healthy subjects with no history of chronic diseases.	12	180 days	Drug: 4-day treatment of antibiotics (500 mg vancomycin, 40 mg gentamycin, and 500 mg of meropenem) daily.	Results of mixed meal testing found no significant changes in postprandial glucose tolerance or secretion of gut-derived incretin hormones following treatment. Abundance of specific gut bacteria was dramatically reduced following short-term course of antibiotics. Conclusion: 4-day course of a cocktail containing 3 antibiotic drugs resulted in acute reversible increase in postprandial levels of PYY but did not significantly alter glucose metabolism.
Park et al. (2020), Korea [36]	RCT	>20 y	34.3	Subjects with healthy and slightly elevated fasting TG levels (<200 mg/dL).	62	14 weeks	Diet: supplementation of probiotic *Lactobacillus plantarum* Q180 (LPQ180) or placebo daily.	LPQ180 ingestion improved PPL metabolism by reducing lipid levels, although the exact mechanism behind this is unknown. Baseline microbiota abundance negatively correlated with lipid marker change. Conclusion: Treatment with LPQ180 significantly decreased LDL-C levels and decreased postprandial maximum concentrations and areas under the curve of TG and chylomicron TG.
Reijnders et al. (2016), Netherlands [37]	RCT	35–70 y	100	Obesity and impaired fasting glucose and/or impaired glucose tolerance	57	7 days	Drug: Comparison of treatment with 1500 mg amoxicillin, 1500 mg vancomycin, or placebo.	Treatment with vancomycin significantly decreased relative abundance of bacteria and diversity in insulin-resistant subjects. Short-term antibiotic treatment had no effect on postprandial forearm substrate metabolism compared to placebo. Non-significant changes in PPGR, PPL, or free fatty acid (FFA) concentrations in either treatment group despite vancomycin-induced changes in gut microbiota.
Ross et al. (2011), Switzerland [38]	Randomised crossover trial	20–50 y	35.3	Healthy subjects with no history of chronic diseases.	17	2 weeks	Diet: Whole grain rich foods (WG) vs. refined grains (RG).	Trend towards decrease in TC and LDL-C in whole grain group in comparison to a refined grain diet after 2 weeks but no difference in fasting TG. No significant differences in plasma HDL-C, glucose, CRP or homocysteine were found between groups. No differences found in PPL or PPGR between the two meal challenges. Large inter-subject variability was noted for total bacteria and specific species analysed. Conclusion: no significant results were reported for either group for PPGR or PPL; however, a trend suggesting a beneficial effect of a diet rich in whole grains on total plasma total cholesterol was noted.
Schutte et al. (2018), Netherlands [39]	Randomised parallel trial	45–70 y	62	Subjects with increased risk of CVD; overweight males and postmenopausal females with mildly elevated levels of plasma total cholesterol (>5 mmol/L).	50	12 weeks	Diet: Whole grain wheat diet (WGW) vs. refined wheat (RW) diet.	Gut microbiota diversity decreased in the refined wheat group compared to the whole grain wheat group. PPL (4 h) increased significantly in the WGW group (*p* = 0.004), resulting in a significant change in overall PPL response between the groups (*p* = 0.020). IHTG levels increased by 49.1% following the 12-wk RW intervention in comparison to the WGW group (*p* = 0.033). Conclusion: A 12-week refined wheat intervention increased IHTG whilst consumption of WGW increased PPL but may prevent substantial accumulation of liver fat via improved hepatic lipid efflux.
Tily et al. (2019), USA [40]	Series of acute experimental studies	≥18 y	~34	Healthy subjects with no history of chronic diseases.	550	2 weeks	Diet: Standardised meal testing to measure individual glycaemic responses.	Metatranscriptomic activity of the gut microbiome is correlated with glycaemic response among adults. Glycaemic response is driven by the properties of an individual and the macronutrient content of foods when measured with multiple diet types and within a multi-ethnic population. Conclusion: Gut microbiome contributes to individual variation in glycaemic response in adults.
Tong et al. (2018), China [28]	RCT	30–65 y	50	Untreated subjects that meet diagnostic criteria for T2D with an elevated waist circumference.)	100	12 weeks	Drug: Comparison of treatment with Chinese herbal formula (AMC) or metformin as a positive control.	Metformin (positive control) and AMC significantly improved 2-h postprandial blood glucose after 12 weeks (*p* < 0.0001). A significant improvement in fasting TG levels was seen in the AMC group in comparison to metformin (*p* < 0.01). Treatment with metformin significantly increased microbiota diversity whereas AMC resulted in a significant decrease in diversity (*p* < 0.05) Conclusion: Treatment with both metformin and AMC significantly improved 2-h postprandial blood glucose and altered gut microbiota structure.
Vetrani et al. (2020), Italy [41]	Randomised parallel trial	40–70 y	42.3	Otherwise healthy subjects at risk of MetS	78	8 weeks	Diet: 4-arm intervention comparing diets of varying levels of long chain n-3 polyunsaturated fatty acids (LCn3) and/or polyphenols (PP) in subjects with MetS risk factors.	Increases in diversity of bacteria was observed after PP rich diets but decreased after consumption of diets low in LCn3 and PP and high in LCn3. Strong positive correlation found between changes in *Atopobium* cluster and postprandial TG and large VLDL-C (AUC at 6 h) (*p* = 0.026 and *p* = 0.009 respectively). Conclusion: Diets rich in polyphenols or LCn3 influenced gut microbiota composition in otherwise healthy subjects at high risk of MetS.
Vors et al. (2020), France [42]	RCT	<75 y	0	Overweight postmenopausal women.	58	4 weeks	Diet: Comparison of milk polar lipid consumption (0, 3 or 5 g-PL/day) or control.	Consumption of milk polar lipids reduced both fasting and postprandial concentrations of TC and TG (*p* < 0.05). Milk polar lipids did not alter SCFA profile or bacterial composition of gut microbiota in post-menopausal subjects. Conclusion: supplementation with milk polar lipids decreased postprandial lipid markers of cardiometabolic risk in overweight, postmenopausal women.
Vrieze et al. (2014), Netherlands [43]	RCT	≥18 y	100	Obese subjects that meet diagnostic criteria for MetS.	20	7 days	Drug: Comparison of treatment with 1500 mg amoxicillin or 1500 mg vancomycin.	Vancomycin reduced faecal microbial diversity by decreasing gram-positive bacteria e.g., Firmicutes (*p* < 0.01). Administration of vancomycin decreased peripheral insulin sensitivity in comparison to the amoxicillin group (*p* < 0.05). Conclusion: treatment with vancomycin significantly decreased gut microbial diversity in comparison to amoxicillin; PPG, TG and FFA concentrations did not change significantly.
Xu et al. (2015), China [44]	RCT	30–65 y	61.5	Newly diagnosed but untreated T2D.	187	12 weeks	Drug: Comparison of high, moderate, or low dose treatment of herbal formula GQD, or placebo.	12-week treatment of GQD significantly improved glycaemic control in subjects with T2D (*p* < 0.001) A non-significant decrease in the mean change of PPG from baseline was observed in the treated groups in comparison to placebo. Results of real-time PCR found that GQD significantly enriched *Faecalibacterium prausnitzii,* which negatively correlated with both postprandial and fasting blood glucose and HbA1c levels. Conclusion: Structural changes of gut microbiota are induced by Chinese herbal formula GQD.
Zeevi et al. (2015), Israel [19]	Series of acute experimental studies	18–70 y	40	Healthy subjects with no previous history of chronic disease.	800	7 days	Diet: Standardised meal testing to measure and predict individual metabolic responses.	High interpersonal variability in post-meal glucose responses. Prediction of glycaemic response using a personalised model framework was found to be superior to common methods used in an independent Israeli cohort. Short-term personalised dietary interventions successfully lowered PPGR. Proteobacteria, Enterobacteriaceae, and KEGG pathways of bacterial chemotaxis, flagellar assembly, and ABC transporters are associated with PPGRs of several standardised meals. Conclusion: Intra-individual variability in PPGRs can be accurately predicted through analysis of individual characteristics and microbiome features. Short-term personalised dietary interventions successfully lower post-meal glucose responses.

**Abbreviations**: BKB, barley kernel-based bread; CHO, carbohydrate; CRP, C-reactive protein; CVD, cardiovascular disease; FFA, free fatty acid; GQD, Gegen Qinlian Decoction; HDL-C, high-density lipoprotein cholesterol; IL-6, interleukin 6; IHTG. intrahepatic triglyceride; LPQ180, Lactobacillus plantarum Q180; LDL-C, low-density lipoprotein cholesterol; LCn3, long chain n-3 polyunsaturated fatty acids; LPS, lipopolysaccharides; MetS, metabolic syndrome; PCR, polymerase chain reaction; PP, polyphenols; PPG, postprandial glucose; PPGR, postprandial glucose response; PPL, postprandial lipids; PPLR, postprandial lipid response; PYY, peptide YY; RS, resistant starch; SCFA, short-chain fatty acids; T2D, type-2 diabetes; TC, total cholesterol; TG, triglycerides; TMAO, trimethylamine n-oxide; VLDL-C, very low-density lipoprotein cholesterol; WWB, white wheat flour bread.

**Table 2:** PICOS (Population, Intervention, Comparison, and Outcomes) table summarising study rationale.

Parameter	Inclusion/Exclusion Criteria
Participants	Adults aged ≥ 18 years.
Interventions	Diet, drug interventions.
Comparisons	Placebo or control group, different diet/intake.
Outcomes	Primary outcomes included presence of both metagenomic and postprandial plasma analysis, namely plasma glucose, lipids, and lipoproteins.
Study design	Randomised controlled or clinical trials with either parallel, crossover or a series of acute experimental studies.

## Supplementary Materials

The following are available online at https://www.mdpi.com/article/10.3390/nu13113887/s1, Table S1: Database Search Strategy.
