# Supplementary material for: The Impact of Microbial Composition on Postprandial Glycaemia and Lipidaemia: A Systematic Review of Current Evidence
Source: Nutrients. 2021 Oct 29;13(11):3887. doi: 10.3390/nu13113887 (PMC8625294; doi:10.3390/nu13113887)
Supplement: Supplementary file 1 [file nutrients-13-03887-s001.zip › nutrients-1413346-Supplementary.pdf]

Table S1. Database Search Strategy.

Database	S#	Search Term
CINAHL+ (EBSCO)	#1	"Microbiome" kw. tx.
	#2	"Microbi*" kw.
	#3	"Gastrointestinal microbiome" kw. tx.
	#4	"Microbiota" [mh] tx.
	#5	"Mycobiome" kw. tx.
	#6	"Gut microbi*" kw. tx.
	#7	"Microbial consortia" kw.
	#8	1 or 2 or 3 or 4 or 5 or 6 or 7
	#9	"Postprandial" ti. ab.
	#10	"Post-prandial" ti. ab.
	#11	"Postprandial period" [mh] tx.
	#12	"Postmeal" tx.
	#13	"Post-meal" tx.
	#14	"Posteating" tx.
	#15	"After feeding" tx.
	#16	"After lunch" tx.
	#17	"After dinner" tx.
	#18	"After meals" tx.
	#19	9 or 10 or 11 or 12 or 13 or 14 or 15 or 16 or 17 or 18
	#20	8 and 19
	#21	"Dyslipidemias" kw. tx.
	#22	"Lipoproteins" [mh] kw. tx.
	#23	"LDL" kw. tx.
	#24	"Low density lipoprotein" tx.
	#25	"Lipid metabolism" kw. ti. ab.
	#26	"Fatty acid metabolism" tx.
	#27	"Lipids" [mh] ti. ab.
	#28	"Dyslipid*" kw. tx.

	#29	"HDL" kw. tx.
	#30	"High density lipoprotein" tx.
	#31	21 or 22 or 23 or 24 or 25 or 26 or 27 or 28 or 29 or 30
	#32	"Glycemia" kw. ti. ab.
	#33	"Glycemic control" [mh] tx.
	#34	"Glucose levels" tx.
	#35	"Glycemic response" ti. tx.
	#36	"Blood glucose" [mh] ti. ab.
	#37	"Glucose metabolism" kw. ab.
	#38	32 or 33 or 34 or 35 or 36 or 37 or 38
	#39	20 and 31
	#40	20 and 38
	#41	39 or 40
	#42	"Random* controlled trial" ab. pt.
	#43	"Controlled clinical trial" ab.
	#44	"Random*" ab. pt.
	#45	"Trial*" ab.
	#46	"Placebo*" ab.
	#47	"Group*" ab.
	#48	42 or 43 or 44 or 45 or 46 or 47 or 48
	#49	41 and 48
	#50	"Humans" [mh] su.
	#51	"Animals" [mh] su.
	52	#49 and #50 not #51

**Database**	**S#**	**Search Term**
PubMed	#1	“Microbiome” kw. tx.
	#2	“Microbi*” kw.
	#3	“Gastrointestinal microbiome” [mm] tx.
	#4	“Microbiota” [mm] tx.
	#5	“Mycobiome” [mm] tx.
	#6	“Gut microbi*” tx.
	#7	“Microbial consortia” [mm]
	#8	1 or 2 or 3 or 4 or 5 or 6 or 7
	#9	“Postprandial” ti/ab.
	#10	“Post-prandial” ti/ab.
	#11	“Postprandial period” [mm] tx.
	#12	“Postmeal” tx.
	#13	“Post-meal” tx.
	#14	“Posteating” tx.
	#15	“After feeding” tx.
	#16	“After lunch” tx.
	#17	“After dinner” tx.
	#18	“After meals” tx.
	#19	9 or 10 or 11 or 12 or 13 or 14 or 15 or 16 or 17 or 18
	#20	8 and 19
	#21	“Dyslipidemias” [mm] tx.
	#22	“Lipoproteins” [mm] tx.
	#23	“LDL” kw. tx.
	#24	“Low density lipoprotein” tx.
	#25	“Lipid metabolism” [mm] ti/ab.
	#26	“Fatty acid metabolism” tx.
	#27	“Lipids” [mm] ti/ab.
	#28	“Dyslipid*” kw. tx.

	#29	"HDL" kw. tx.
	#30	"High density lipoprotein" tx.
	#31	21 or 22 or 23 or 24 or 25 or 26 or 27 or 28 or 29 or 30
	#32	"Glycemia" kw. ti/ab.
	#33	"Glycemic control" tx.
	#34	"Glucose levels" tx.
	#35	"Glycemic response" ti/ab. tx.
	#36	"Blood glucose" [mm] ti/ab.
	#37	"Glucose metabolism" kw. ti/ab.
	#38	32 or 33 or 34 or 35 or 36 or 37
	#39	20 and 31
	#40	20 and 38
	#41	39 or 40
	#42	"Random* controlled trial" ti/ab. pt.
	#43	"Controlled clinical trial" ti/ab. pt.
	#44	"Random*" ti/ab.
	#45	"Trial*" ti/ab.
	#46	"Placebo*" ti/ab.
	#47	"Group*" ti/ab.
	#48	36 or 37 or 38 or 39 or 40 or 41
	#49	41 and 48
	#50	"Humans" [mt]
	#51	"Animals" [mt]
	#52	#49 and #50 not #51

Database	S#	Search Term
Cochrane Central	#1	"Microbiome" kw. tx.
	#2	"Microbi*" kw.
	#3	"Gastrointestinal microbiome" [mh] tx.
	#4	"Microbiota" [mh] tx.
	#5	"Mycobiome" [mh] tx.
	#6	"Gut microbi*" tx.
	#7	"Microbial consortia" [mh]
	#8	1 or 2 or 3 or 4 or 5 or 6 or 7
	#9	"Postprandial" ti. ab.
	#10	"Post-prandial" ti. ab.
	#11	"Postprandial period" [mh] tx.
	#12	"Postmeal" tx.
	#13	"Post-meal" tx.
	#14	"Posteating" tx.
	#15	"After feeding" tx.
	#16	"After lunch" tx.
	#17	"After dinner" tx.
	#18	"After meals" tx.
	#19	9 or 10 or 11 or 12 or 13 or 14 or 15 or 16 or 17 or 18
	#20	8 and 19
	#21	"Dyslipidemias" [mh] tx.
	#22	"Lipoproteins" [mh] kw. tx.
	#23	"LDL" kw. tx.
	#24	"Low density lipoprotein" tx.
	#25	"Lipid metabolism" [mh] ti. ab.
	#26	"Fatty acid metabolism" tx.
	#27	"Lipids" [mh] ti. ab.
	#28	"Dyslipid*" kw. tx.

	#29	“HDL” kw. tx.
	#30	“High density lipoprotein” tx.
	#31	21 or 22 or 23 or 24 or 25 or 26 or 27 or 28 or 29 or 30
	#32	“Glycemia” kw. ti. ab.
	#33	“Glycemic control” tx.
	#34	“Glucose levels” tx.
	#35	“Glycemic response” ti. tx.
	#36	“Blood glucose” [mh] ti. ab.
	#37	“Glucose metabolism” kw. ab.
	#38	32 or 33 or 34 or 35 or 36 or 37
	#39	20 and 31
	#40	20 and 38
	#41	39 or 40
	#42	“Random* controlled trial” ab. pt.
	#43	“Controlled clinical trial” ab. pt.
	#44	“Random*” ab. pt.
	#45	“Trial*” ab.
	#46	“Placebo*” ab.
	#47	“Group*” ab.
	#48	36 or 37 or 38 or 39 or 40 or 41
	#49	41 and 48
	#50	“Humans" [mh] ti,ab,kw.
	#51	“Animals” [mh] ti, ab, kw.
	#52	#49 and #50 not #51

Database	S#	Search Term
Web of Science	#1	"Microbiome" ts.
	#2	"Microbi*" ak.
	#3	"Gastrointestinal microbiome" ts.
	#4	"Microbiota" ts.
	#5	"Mycobiome" ak.
	#6	"Gut microbi*" ts.
	#7	"Microbial consortia" ak.
	#8	1 or 2 or 3 or 4 or 5 or 6 or 7
	#9	"Postprandial" ti. ab.
	#10	"Post-prandial" ti. ab.
	#11	"Postprandial period" ak.
	#12	"Postmeal" ts.
	#13	"Post-meal" ts.
	#14	"Posteating" ts.
	#15	"After feeding" ts.
	#16	"After lunch" ts.
	#17	"After dinner" ts.
	#18	"After meals" ts.
	#19	9 or 10 or 11 or 12 or 13 or 14 or 15 or 16 or 17 or 18
	#20	8 and 19
	#21	"Dyslipidemias" ak.
	#22	"Lipoproteins" ak.
	#23	"LDL" ak.
	#24	"Low density lipoprotein" ts.
	#25	"Lipid metabolism" ti. ab.
	#26	"Fatty acid metabolism" ts.
	#27	"Lipids" ti. ab.
	#28	"Dyslipid*" ak.

	#29	“HDL” ak.
	#30	“High density lipoprotein” ts.
	#31	21 or 22 or 23 or 24 or 25 or 26 or 27 or 28 or 29 or 30
	#32	“Glycemia” ak. ti. ab.
	#33	“Glycemic control” ts.
	#34	“Glucose levels” ts.
	#35	“Glycemic response” ti. ts.
	#36	“Blood glucose” ti. ab.
	#37	“Glucose metabolism” ti. ab.
	#38	32 or 33 or 34 or 35 or 36 or 37
	#39	20 and 31
	#40	20 and 38
	#41	39 or 40
	#42	“Random* controlled trial” ab. ak.
	#43	“Controlled clinical trial” ab. ak.
	#44	“Random*” ab.
	#45	“Trial*” ab.
	#46	“Placebo*” ab.
	#47	“Group*” ab.
	#48	36 or 37 or 38 or 39 or 40 or 41
	#49	41 and 48
	#50	" Humans" ts.
	#51	“Animals” ts.
	#52	#49 and #50 not #51
